# Medullary Thyroid Carcinoma and Associated Endocrinopathies in Slovenia from 1995 to 2021

**DOI:** 10.3390/life12071091

**Published:** 2022-07-21

**Authors:** Sara Milicevic, Mateja Krajc, Ana Blatnik, Barbara Peric

**Affiliations:** 1 Institute of Oncology Ljubljana, Zaloska 2, 1000 Ljubljana, Slovenia; smilicevic@onko-i.si (S.M.); mkrajc@onko-i.si (M.K.); ablatnik@onko-i.si (A.B.); 2 Faculty of Medicine, University of Ljubljana, Zaloska 2, 1000 Ljubljana, Slovenia

**Keywords:** medullary thyroid carcinoma, multiple endocrine neoplasia, *RET* pathogenic variant, pheochromocytoma, primary hyperparathyroidism

## Abstract

Background: Medullary thyroid cancer (MTC) is a rare endocrine tumour that is sporadic in 75% of cases and occurs as a part of inherited cancer syndromes in approximately 25% of cases. The aim of this study was to determine the frequency and type of *RET* pathogenic variants (PVs) in the Slovenian MTC patient population diagnosed between 1995 and 2021 and to elucidate the full range of associated endocrinopathies. Methods: A retrospective analysis of medical records of 266 MTC patients and their relatives seen in a tertiary centre between 1995 and 2021 was performed. Sequence analysis of exons 10, 11, 13, 14, 15, and 16 of the *RET* gene was analysed in most patients using Sanger sequencing. From 2017, the entire sequence of RET gene was analysed in most patients using targeted next-generation sequencing. Results: Germline PVs in the *RET* proto-oncogene were identified in 21.6% probands from 21 different MTC families. Of their tested relatives, 65% (67/103) were *RET*-positive and 35% (36/103) were *RET*-negative. PVs were detected in codon 618 and codon 634 in 28.6%, and in codon 790 in 23.8%. The *RET*-positive group consisted of 52 MTC patients, 13 patients with C cell hyperplasia and 2 individuals with neither. Associated endocrinopathies were diagnosed in 8/21 families: primary hyperparathyroidism (PHPT) in six families and pheochromocytoma (PHEO) in five families. In 62% of *RET*-positive families (13/21), no associated endocrinopathies were diagnosed. PHEO was most commonly associated with C634R (6/13) and PHPT with C634R (4/7). Hirschsprung’s disease appeared in one patient with *RET* PV in codon 618. Based on data from the Cancer Registry of Republic of Slovenia, only individual cases of common cancers with well understood environmental risk factors were discovered; lung cancer in 2/21 of families, papillary thyroid cancer in 3/21 of families, cutaneous melanoma in 2/21 of families, cervical cancer in 1/21 families, and lymphoma in 1/21 families. Conclusions: Analysis of prospectively collected MTC cases during a 27-year period revealed that 21.6% of Slovenian patients are *RET* PV carriers. Sixty-two percent of families had none of the associated endocrinopathies, confirming the thesis that FMTC is the most common presentation. This could suggest using risk-stratified management approaches when screening for PHEO and PHPT in *RET* PV carriers. However, more studies are needed to evaluate potential genetic risk modifiers as well as safety, improved quality of life, and medical cost reduction in the case of a patient-oriented approach.

## 1. Introduction

Medullary thyroid cancer (MTC) is a rare endocrine tumour. In most cases, it is sporadic, but it can also occur as part of inherited cancer syndromes, in approximately 25% of cases [1]. An autosomal-dominant hereditary syndrome called multiple endocrine neoplasia type 2 (MEN2) is caused by activating pathogenic variants (PVs) in the rearranged during transfection gene (*RET*) on chromosome 10q11.2 [2]. It encodes a tyrosine kinase involved in the development of the parathyroid glands, adrenal medulla, parafollicular C cells, and enteric ganglia, and it is associated with MTC, pheochromocytoma (PHEO), primary hyperparathyroidism (PHTH), Hirschsprung’s disease (HSCR), and other characteristic phenotypic features such as mucosal neuromata, marfanoid habitus, and corneal nerve thickening [3]. According to the clinical manifestations, hereditary MTC may be classified into three clinically distinct subtypes: MEN2A, MEN2B, and familial medullary thyroid carcinoma (FMTC) [1].

Soon after the introduction of *RET* gene testing, it was recognized that the phenotype may differ according to the location of the PV [4,5]. In apparently sporadic patients with MTC, PVs were most often found in non-cysteine codons of the *RET* genes, since PVs in this region are predictive of FMTC phenotype [6]. Conversely, a large proportion of patients presenting with PHEO and/or PHPT have PVs in the cysteine-rich extracellular domain, particularly in exons 10 and 11. However, aggressiveness of the disease and accompanied endocrinopathies might also vary depending on the specific amino acid substitutions [7].

According to the American Thyroid Association guidelines for the management of MTC, yearly screening for PHEO and PHTH should begin by age 11 years for *RET* PV carriers classified in the ATA high and highest categories and by age 16 years for *RET* PV carriers in the moderate category [8]. However, the NCCN guidelines propose annually screening for PHEO (MEN 2A and MEN 2B) and PHTH (MEN 2A), while for carriers of *RET* PVs in codons 768, 790, 804, or 891, less frequent screening may be appropriate [9].

For rare diseases where there is heterogeneity in genotype and phenotype, and an age-related penetrance, correct estimation of risk for disease manifestations is challenging. The aim of this study was to evaluate the frequency and type of *RET* PVs in the Slovenian MTC patient population diagnosed between 1995 and 2021, to elucidate the full range of associated endocrinopathies together with possible other cancers, and to compare our results with other population reports.

## 2. Methods

A retrospective analysis of 266 MTC patients and their relatives seen at the Institute of Oncology Ljubljana (IOL) between 1995 and 2021 was undertaken. IOL is a national referral centre for diagnostics and treatment of thyroid cancer, where genetic counselling and testing is also available. In Slovenia, genetic counselling and testing are offered to all patients diagnosed with MTC and to patients’ family members in cases with confirmed *RET* PV. If a germline *RET* PV in an asymptomatic person was detected, prophylactic total thyroidectomy was proposed.

Two different data sources were used, the hospital-based registry of patients with MTC and the Cancer Registry of Republic of Slovenia. The former includes prospectively collected epidemiologic data and molecular genetic data from patients and relatives who underwent genetic testing since the introduction of *RET* gene testing in 1995, while the latter is one of the oldest population-based cancer registries in the Europe that has been gathering data on all cancer types and issuing annual reports since 1950.

All patients gave their written consent before genetic counselling and testing. Sequence analysis of exons 10, 11, 13, 14, 15, and 16 of the *RET* gene was analysed in most patients using Sanger sequencing, as previously described [10]. From 2017, the entire sequence of RET gene was analysed in most patients using targeted next-generation sequencing.

To examine the frequency of associated endocrinopathies, we expanded our observation from the original 208 MTC patients to 7 family members diagnosed with MTC before 1995 and 15 asymptomatic *RET* PV carriers belonging to one of 21 *RET*-positive families. To determine whether there was any association between *RET* PVs and other cancers, we analysed data from the Cancer Registry of Republic of Slovenia.

A literature search on MEDLINE (via Pubmed) was conducted. Search terms “medullary thyroid carcinoma, medullary thyroid cancer, *RET* gene, multiple endocrine neoplasia type2, and MEN2” were used. Retrospective studies reporting frequencies of PHEO and PHPT in association with different *RET* PVs, written in English and published between 2012 and 2022, were included. Additionally, manual searches in reference lists of the relevant articles was also performed.

For reporting specific phenotypes or genotypes, total numbers of cases and percentages were used. For continuous variables, the mean (±SD) was used, unless otherwise specified. Continuous variables were analysed using an independent *t*-test. The statistical analysis was performed with IBM SPSS Statistics, version 27. 

## 3. Results

### 3.1. Study Sample

Altogether 266 individuals were included in the analysis. Among 208 patients diagnosed with MTC between 1995 and 2021, 110 (52.9%) patients were female. In total, 45 (21.6%) patients were *RET*-positive, while 134 (64.4) patients were *RET*-negative. In 10 (4.8%) patients, a variant of unknown significance (VUS) was found. Nineteen (9.1%) patients did not undergo genetic testing, or the results of testing were not yet available. The mean age at the time of diagnosis was lower in the group of the *RET*-positive patients than in *RET*-negative patients (42.0 ± 19.0 years vs. 59.8 ± 13.9 years, *p* < 0.001).

### 3.2. Genetic Testing Results

With *RET* genetic testing, a total of 21 *RET*-positive families were diagnosed and followed on IOL between 1995 and 2021. On average, 4.9 individuals per family were tested, with 103 tested family members altogether. Among those individuals, 65% (67/103) were *RET*-positive and 35% (36/103) were *RET*-negative. After additional data inclusion, the *RET*-positive group consisted of 52 MTC patients (45 patients diagnosed with MTC between 1995 and 2021 and 7 patients before 1995), 13 patients with C cell hyperplasia, and 2 individuals without either.

Overall, 10 different *RET* PVs and 6 different VUS were identified. Recurrent PVs were detected in codon 618 and codon 634 in 28.6% (6/21), in codon 790 in 23.8% (5/21), and in codon 804 in 9.5% (2/21) of *RET*-positive families. PVs of codons 891 and 918 were rare (1/21, 4.8%).

### 3.3. Associated Endocrinopathies

As seen in Table 1, associated endocrinopathies were diagnosed in only 8/21 families: HPTH in six families and PHEO in five families. In 62% of *RET*-positive families (13/21), no associated endocrinopathies were diagnosed. In 13 patients with PHEO, the most common *RET* PV was C634R (6/13), followed by C634Y (4/13), C634G (c.1900T > G) (2/13), and C618F (c.1853G > T) (1/13). Among seven cases with HPTH, the C634R *RET* PV was detected in four cases, L790F (c.2370G > T) in two cases, and C634G (c.1900T > C) in one case. HSCR appeared in one patient with *RET* PV in codon 618.

### 3.4. Comparison with Other Studies and the American Thyroid Association Guidelines

We compared our results to the data of the American Thyroid Association guidelines for the management of MTC [8]. We also examined studies reporting the incidence of accompanied endocrinopathies in other populations. The comparison of the relationships among common *RET* PVs and percentages of accompanied endocrinopathies is showed in Table 1.

### 3.5. Other Cancers

An additional analysis of *RET*-positive families showed that lung cancer was diagnosed in 2/21 of families, papillary thyroid cancer (PTC) in 3/21 of families, cutaneous melanoma in 2/21 of families, cervical cancer in 1/21 of families, and lymphoma in 1/21 of families. No other cancers were diagnosed in *RET*-positive families.

## 4. Discussion

We present a population-based analysis of *RET*-associated endocrinopathies, which have rarely been performed in the past.

### 4.1. Genetic Testing Results

This national long follow-up retrospective study showed that in 62% of *RET*-positive families no associated endocrinopathies were observed. Comparing our results with data reported in the ATA guidelines, we observed lower frequencies of PHEO and PHPT in almost all *RET* PVs except for the carriers of codon 634 PVs [8]. Higher PHEO and PHPT frequencies observed in our population could be a result of a long follow-up and higher number of C634R carriers. Namely, different penetrances of PHEO and PHPT were reported in association with different amino acid substitutions in *RET* codon 634, with the highest penetrance in C634R, followed by C634Y, C634W, C634G, and C634 [11,13,18]. However, we observed higher frequencies of accompanied endocrinopathies not only in C634G (PHEO 2/2, 100% and PHPT 1/2, 50.0%) but also in C634R (PHEO 6/8, 75.0% and PHPT 4/8, 50.0%) as compared with C634Y (PHEO 4/7, 57.1% and PHPT 0/7, 0%).

### 4.2. Associated Endocrinopathies and Comparison with Other Studies and the American Thyroid Association Guidelines

The frequency of observed PHPT (10.5%) in L790F carriers in our study was similar to the frequency reported in the ATA guidelines [8]. No other associated endocrinopathies were diagnosed for carriers of PVs in exons 13–15. These results were in accordance with previous reported studies, where absence or low incidences of associated endocrinopathies were reported [6,11,16]. Unlike other studies, we did not observe PHEO in association with MEN2B syndrome [11,14,16]. This could be explained by the fact that only one carrier of M918T, who was 15 years old at the time of data analysis, was included in our study. The patient was found to be a carrier of a de novo M918T PV.

Recently, a comprehensive review of the distribution and frequency of *RET* PVs in patients with MTC from the global perspective was published. It reported a wide spectrum of *RET* variants and the list of variants is continually updated [7]. In many countries, PVs in codon 634 are the most prevalent. Relative frequencies between 29% and 36% are reported in major European series, but even a 67.6% frequency was reported in the EUROMEN multicentre study [6,7,11,12,15,19]. In our population, the most frequent *RET* PVs occurred not only in codon 634 but also in codon 618, both in 28.5%. Thus, the frequency of PVs in codon 618 in our study is three- to four-fold higher than that reported in other European countries [7]. We also observed much higher frequencies of L790P than in other European countries (23,8% vs. 11.6% in Germany). As expected, lower frequencies of variants V804M (9.5%) and S891A (4.8%) were observed. Those variants have been described as founder variants in northern Italy and Sardinia and are rare elsewhere [7,20,21]. These findings demonstrate national specific distribution of *RET* PVs.

Our study supports the idea of ATA guideline revisions regarding yearly screening for PHEO and PHPT in all *RET* PVs carriers. Instead, family-oriented screening could be implemented based on risk evaluation and specific *RET* variants [8]. While *RET* PVs in codons 634 and 918 are reported to be strongly associated with PHEO and PHPT, the association of PHEO and PHPT with non-cysteine *RET* PVs is rare. Consequently, annual screening of methanephrines and parathyroid hormone seems to be cost ineffective and might negatively affect individuals’ quality of life. Some authors have proposed modification of the follow-up protocol for FMTC with non-cysteine *RET* PVs, where screening for PHEO and PHPT could be performed every five years or in the case of clinical suspicion [6].

### 4.3. Other Cancers

Strong correlations of *RET* PVs with other cancers has not been observed [7]. Interestingly, Kovac et al. recently reported about a MEN2 family whose 32-year-old member developed osteosarcoma two years after resection of MTC. Germline *RET* S891A PV was found. To determine if both disorders had a common genetic background, they analysed next-generation sequencing data from germline DNA of 336 patients with osteosarcoma. In addition to the S891A, they detected *RET* PV in codon 620 in two patients and one in codon 603 [22]. However, considering the fact there is no functional data supporting the association between *RET* PVs and osteosarcoma, such findings should be interpreted with due caution.

With PTC being the most common thyroid cancer, MTC with concurrent PTC has been described in up to 19% of cases [23]; 9.1% of patients had *RET* PV in exon 13 or 14, while PTC has rarely been reported in patients with cysteine PVs [17,24,25]. PTC has also beemn described in the presence of V804M PV [26]. In our series, PTC was found in 2/19 (10.5%) of patients with PV L790F, 1/10 of patients with C618F, and 1/8 of patients with C634R. The reports in the literature suggest that concurrent occurrence of distinct tumours in the same thyroid gland are probably a coincidental finding [23].

### 4.4. Limitations of the Study

Even though our study is a population-based analysis with a long-term follow-up, it has some limitations. The main limitation is the small number of *RET* PVs carriers because of a small population size and low compliance of family members resulting in an incomplete number of tested individuals. In addition, exons 5, 8, and 9 were not yet analysed in most patients which may have led to underestimation of the frequency of *RET* PVs in the Slovenian population. Our retrospective analysis was performed in line with other published studies but heterogeneity of observations and methods of patient management over multiple centres can make it difficult to compare the results. As a case in point, some authors only reported accompanying endocrinopathies in patients with *RET* PV and MTC, excluding *RET* pathogenic variant carriers with no MTC from their analyses.

## 5. Conclusions

A retrospective analysis of prospectively collected MTC cases during a 27-year periods revealed that 21.6% of Slovenian patients are *RET* PV carriers. Associated endocrinopathies were diagnosed in 38.1% of families. Sixty-two percent of families had none of the associated endocrinopathies, confirming that FMTC is the most common disease presentation. This suggests that we can usher in the era of personalized medicine and start to use a risk-stratified genotype-phenotype correlation management approach for screening PHEO and PHPT in *RET* PV carriers. For FMTC with PVs in codons 790, 804, and 891, PHEO and PHTH screening every five years or only in case of clinical suspicion may be appropriate. However, more studies are needed to evaluate potential genetic risk modifiers as well as safety, improved quality of life, and medical cost reduction in the case of a patient-oriented approach.

## Figures and Tables

**Table 1:** Comparison of frequencies of observed endocrinopathies in patients with common *RET* PVs in our population, eight major published studies, and the American Thyroid Association guidelines.

Exon	*RET* PV (MTC Risk)	Endocrinopathies	Slovenia No. of Patients (%)	ATA [8]	Germany [11]	France [12]	Spain [13]	Denmark [14]	UK [15]	Italy [6]	Brazil * [16]	China [17]
10	C618F/R/S (moderate)	HPTH	0/22 (0%)	≈10%	NA	NA	NA	NA	NA	NA	1/11 (9.1%)	NA
		PHEO	1/22 (4.5%)	10–20%	9/25 (36.0%)	NA	NA	NA	NA	NA	3/11 (27.3%)	0/13 (0%)
		HSCR	1/22 (4,5%)	Positive occurrence	NA	NA	NA	NA	NA	0%	0/11 (0%)	NA
11	C634F/G/R/S/W/Y (high)	HPTH	5/17 (29.4%)	≈20%	NA	NA	9/199 (4.5%)	NA	NA	NA	46/262 (17.6)	NA
		PHEO	12/17 (70.6%)	≈50% Increased with age	56/120 (46.0%)	55/112 (49.1%)	85/199 (42.7%)	NA	20/31 (64.5 %)	NA	95/262 (36.3%)	21/46 (45.7%)
13	L790F (moderate)	HPTH	2/19 (10.5%)	≈10%	NA	NA	NA	NA	NA	0%	0/4 (0%)	NA
		PHEO	0/19 (0%)	/	NA	NA	NA	NA	NA	0%	0/4 (0%)	0/2 (0%)
14	V804M (moderate)	HPTH	0/3 (0%)	≈10%	NA	NA	NA	NA	NA	0%	1/32 (3.1%)	NA
		PHEO	0/3 (0%)	≈10%	NA	NA	NA	NA	NA	0%	0/32 (0%)	NA
15	S891A (moderate)	HPTH	0/5 (0%)	≈10%	NA	NA	NA	NA	NA	0%	0/23 (0%)	NA
		PHEO	0/5 (0%)	≈10%	NA	NA	NA	NA	NA	0%	0/23 (0%)	NA
16	M918T (highest)	HPTH	0/1 (0%)	/	NA	/	NA	NA	NA	NA	1/32 (3.1%)	NA
		PHEO	0/1 (0%)	≈50%	7/17 (41%)	NA	NA	3/7 (42.9%)	NA	NA	12/32 (37.5%)	NA

* Only patients with the diagnosis of MTC included (asymptomatic *RET* PV carriers were not included). NA—not available; PV—pathogenic variants; PHEO—pheochromocytoma; HPTH—primary hyperparathyroidism; HSCR—Hirschsprung’s disease.

## Data Availability

The data presented in this study are available on request from the corresponding author. The data are not publicly available due to national data privacy legislation.
